# Cloning and characterization of mr-s, a novel SAM domain protein, predominantly expressed in retinal photoreceptor cells

**DOI:** 10.1186/1471-213X-6-15

**Published:** 2006-03-16

**Authors:** Tatsuya Inoue, Koji Terada, Akiko Furukawa, Chieko Koike, Yasuhiro Tamaki, Makoto Araie, Takahisa Furukawa

**Affiliations:** 1 Osaka Bioscience Institute; 6-2-4 Furuedai, Suita, Osaka 565-0874, Japan; 2 PRESTO, Japan Science and Technology Agency; 4-1-8 Honcho, Kawaguchi, Saitama, Japan; 3 Department of Ophthalmology, Tokyo University School of Medicine; 7-3-1 Hongo, Bunkyo-ku, Tokyo 113-0033, Japan; 4 Department of Ophthalmology, Osaka University Medical School; Yamadaoka, Suita, Osaka 565-0871, Japan

## Abstract

**Background:**

Sterile alpha motif (SAM) domains are ~70 residues long and have been reported as common protein-protein interaction modules. This domain is found in a large number of proteins, including Polycomb group (PcG) proteins and ETS family transcription factors. In this work, we report the cloning and functional characterization of a novel SAM domain-containing protein, which is predominantly expressed in retinal photoreceptors and the pineal gland and is designated mouse *mr-s *(major retinal SAM domain protein).

**Results:**

*mr-s *is evolutionarily conserved from zebrafish through human, organisms through which the mechanism of photoreceptor development is also highly conserved. Phylogenetic analysis suggests that the SAM domain of mr-s is most closely related to a mouse polyhomeotic (ph) ortholog, Mph1/Rae28, which is known as an epigenetic molecule involved in chromatin modifications. These findings provide the possibility that mr-s may play a critical role by regulating gene expression in photoreceptor development. *mr*-*s *is preferentially expressed in the photoreceptors at postnatal day 3–6 (P3-6), when photoreceptors undergo terminal differentiation, and in the adult pineal gland. Transcription of *mr-s *is directly regulated by the cone-rod homeodomain protein Crx. Immunoprecipitation assay showed that the mr-s protein self-associates mainly through the SAM domain-containing region as well as ph. The mr-s protein localizes mainly in the nucleus, when mr-s is overexpressed in HEK293T cells. Moreover, in the luciferase assays, we found that mr-s protein fused to GAL4 DNA-binding domain functions as a transcriptional repressor. We revealed that the repression activity of mr-s is not due to a homophilic interaction through its SAM domain but to the C-terminal region.

**Conclusion:**

We identified a novel gene, *mr-s*, which is predominantly expressed in retinal photoreceptors and pineal gland. Based on its expression pattern and biochemical analysis, we predict that *mr*-*s *may function as a transcriptional repressor in photoreceptor cells and in pinealocytes of the pineal gland.

## Background

In the development of the mammalian retina, a diverse range of cell types is generated from a pool of multipotent retinal progenitor cells. Among these cell types, photoreceptors account for over 70% of all cells in the retina. In vertebrates, there are two classes of photoreceptors, rods and cones. Rods are sensors of dim light, while cones function in bright light and are responsible for color vision. Phototransduction, a series of signal amplifications detecting a single photon of light, is initiated by the capture of light with 11-cis-retinal, a chromophore bound by the opsin proteins: rhodopsin in rods and cone opsins in cones. The proteins that carry out phototransduction are located in an elaborate and highly specialized membranous structure, the outer segment. The outer segment appears to be relatively fragile, degenerating in response to many environmental and/or genetic perturbations. In the rodent retina, the production of specific cell types during development progresses in a general order [1,2]. Rod photoreceptor generation peaks around the time of birth. Cone photoreceptors, ganglion cells, horizontal cells and amacrine cells are generated earlier, while Müller glia and bipolar cells are generated later. This production of different cell types at different times appears to derive from differences in the intrinsic properties of progenitor cells involved in the transcription or chromatin modification. Recent studies identified several important transcription factors of photoreceptor development [3-7]. Two *Otx *family homeobox genes, *Otx2 *and *Crx*, play essential roles in early cell fate determination and terminal differentiation of photoreceptors [3,8,9]. In the absence of Otx2 function, differentiating photoreceptor cells are converted to amacrine-like neurons [9]. *Crx*, a downstream target of Otx2, controls the transcription of various photoreceptor cell-specific genes and is essential for the formation of outer segments, synaptic terminals, and phototransduction pathways [8,10]. *Crx *transcripts begin to be expressed in developing photoreceptors at embryonic day 12.5 (E12.5) in the mouse and a strong upregulation of *Crx *transcription is apparent across the differentiating photoreceptors at postnatal day 6 (P6). Photoreceptor cells in the *Crx *knockout (KO) mice exhibit a dramatic reduction of many photoreceptor molecules including visual pigments and develop neither photoreceptor outer segments nor a synaptic terminus [8,10]. In addition, mutations of human *CRX *have been demonstrated to be associated with three types of photoreceptor diseases: autosomal dominant cone-rod dystrophy 2, autosomal dominant-type retinitis pigmentosa, and Leber's congenital amaurosis (LCA) [11-14]. While *Otx2 *and *Crx *control general photoreceptor development, three other transcription factors, *TRβ2*, *Nrl*, and *Nr2e3 *regulate the specification of photoreceptor cell types [4,5,7].

SAM domains (also known as Pointed, HLH, or SPM domains) are ~70 residues long and have been reported as common protein-protein interaction modules [15-17]. This domain is found in a large number of proteins, including Polycomb group (PcG) proteins [18], serine threonine kinases [19], Eph family receptor tyrosine kinases [20], the p73 tumor suppressor [21], the RNA-binding protein Smaug [22], diacylglycerol kinases [23,24], yeast mating type signaling proteins [19,25] and ETS family transcription factors [26,27]. The PcG proteins are transcriptional repressors that maintain gene silencing during development [28-30]. In mammals, PcG proteins are also implicated in *Hox *gene regulation. Their biological activity lies in stable silencing of specific sets of genes through chromatin modifications. A member of polycomb repressive complex 1 (PRC1), ph, contains a SAM domain at the C-terminal, and PRC1 complex is known to form helical, head-to-tail polymers through its SAM domain [31]. These polymeric structures mediate the formation of a higher order chromatin structure and possibly stabilize the repressed state of *Hox *genes.

In this study, we identified a novel gene, *mr-s*, which encodes a SAM domain-containing protein. The SAM domain of mr-s is most closely related to that of ph mouse ortholog, MPH1/Rae28. *mr-s *is predominantly expressed in retinal photoreceptors when they undergo terminal differentiation, and adult pineal gland. The expression of *mr-s *is directly regulated by Crx. Moreover, mr-s is localized in the nucleus and can self-associate through its SAM domain-containing region. We also found that mr-s protein fused to GAL4 DNA-binding domain functions as a transcriptional repressor. These findings suggest that *mr-s *functions as a member of a transcriptional repressor complex in retinal photoreceptor development.

## Results

### Cloning of mouse *mr-s*

**Figure 1:**
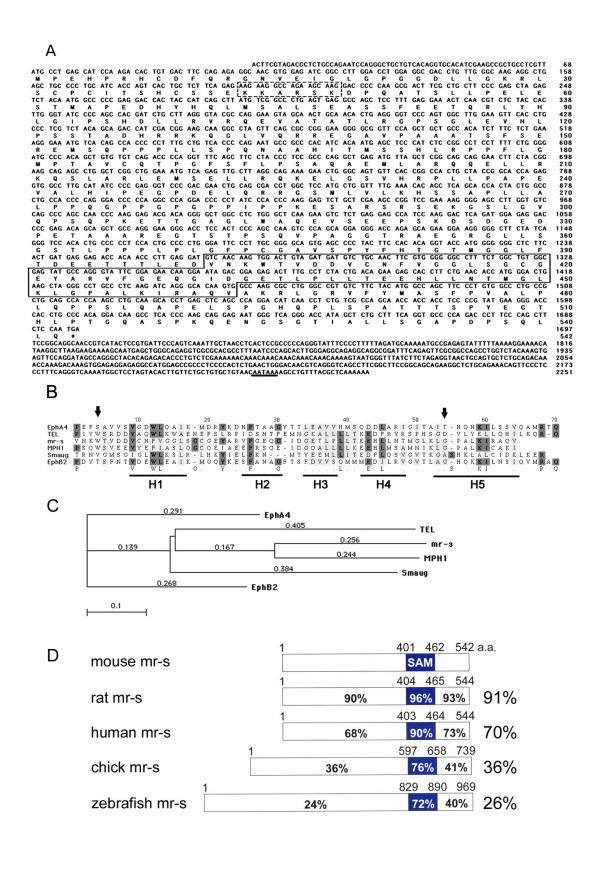
***mr-s *nucleotide and amino acid sequences. **(A) *mr-s *nucleotide and amino acids sequences. Boxed amino acids are the SAM domain sequence and the dashed box indicates a putative nuclear localization signal. The underline indicates a putative polyadenylation termination signal. (B) Alignment of SAM domain sequences for SAM domain-containing proteins. The five alpha helices are marked H1-H5. Conserved amino acid residues are shown with a dark shadow and functionally similar residues are shown with a light shadow. The sites that were targeted for mutagenesis are indicated by arrows. (C) Phylogenetic tree of SAM domain-containing proteins. Amino acid sequences were analyzed by the neighbor-joining method in MacVector 7.2. Branch lengths reflect the mean number of substitutions per site. (D) Schematic comparison of the amino acid sequences for mouse, rat, human, chick and zebrafish mr-s proteins. The percent similarity of the SAM domains and other regions to the corresponding regions of the mouse protein is shown. Overall sequence similarity with the mouse protein is shown on the right.

In order to identify novel mouse genes preferentially expressed in the developing retina, we screened the National Institute for Biotechnology Information (NCBI) database, UniGene, using Digital Differential Display (DDD) and found EST fragments which are frequently present in mouse retinal cDNA libraries. We found that one clone in these cDNAs encodes a protein containing a SAM domain related to that of polyhomeotic protein. A PCR fragment corresponding to this mouse clone was used to screen a mouse P0-P3 retinal cDNA library to obtain a full-length cDNA clone. Sequence analysis showed that this cDNA was a novel gene encoding a SAM domain-containing protein. We referred to this protein as mr-s (major retinal SAM domain protein). As shown in Fig. 1A, a translation initiation codon is present in the same open reading frame as the SAM domain. This initiation site shows similarity to the consensus sequence proposed by Kozak [32] including the presence of the highly conserved purine at position -3. The stop codon of the predicted mr-s protein is also indicated in Fig. 1A. The amino acid sequence of the SAM domain of mr-s protein (Fig. 1A, boxed sequence) exhibits homology with SAM domains of EphB2, EphA4, MPH1, TEL and Smaug (Fig. 1B). By phylogenetic analysis, the SAM domain of mr-s is most closely related to that of Mph1/Rae28, a mouse homolog of ph (Fig. 1C). Mouse mr-s protein is conserved in rat, human, chick and zebrafish, which display 91%, 70%, 36% and 26% identity with mouse mr-s, respectively (Fig. 1D). The SAM domains of rat, human, chick and zebrafish mr-s protein are highly conserved and display 96%, 90%, 76% and 72% identity with the SAM domain of mouse mr-s protein. The chromosomal localizations of mouse and human *mr-s *genes were determined by searching the mouse and human genome databases (NCBI), respectively. Mouse *mr-s *is mapped to chromosome 4E2, and human *MR-S *is mapped to chromosome 1p36.33. LCA is the most common cause of inherited childhood blindness. Human *MR-S *maps in the vicinity region of the LCA9, recently identified as a new locus for LCA [33].

### Expression of *mr-s *in the developing retina and the pineal gland

**Figure 2:**
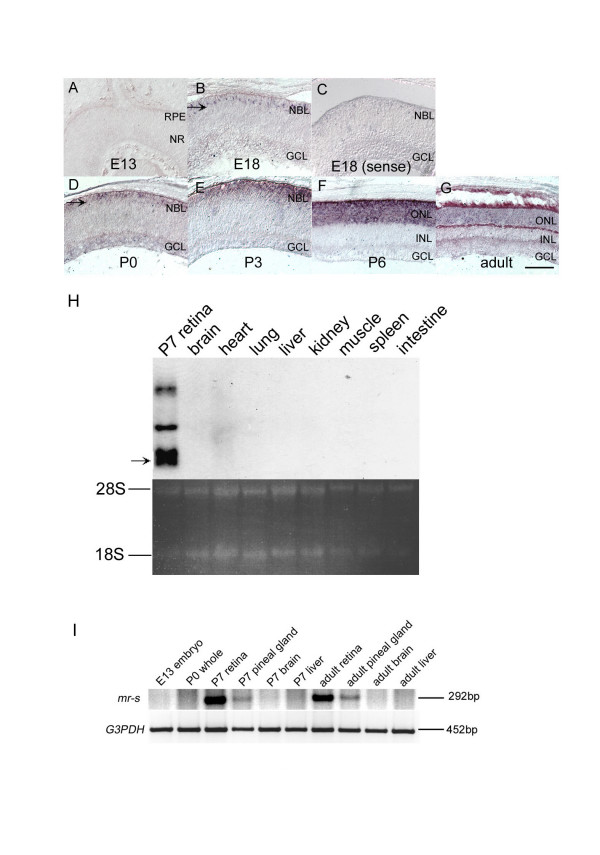
***mr-s *expression in developing mouse retina and pineal gland. **(A-G) *mr-s *expression during development of the mouse retina. The *in situ *hybridization signal of *mr-s *was not detected at E13 (A). The signal (arrow) was first detected in the outer aspect of NBL at E18 (B). A strong *mr-s *signal was detected in outer layer of the retina at P3-P6, and then the signal decreased in the adult retina (E-G). Control with the sense probe in E18 retina is shown (C). Scale bar, 100 μm. (H) Northern blot analysis of *mr-s *expression in adult mouse organs. The arrow corresponds to 2.2kb *mr-s *transcript. (I) RT-PCR analysis of total RNAs extracted from E13 whole embryo, P0 whole body (except for the eye), P7 retina, P7 pineal gland, P7 brain, P7 liver, adult retina, adult pineal gland, adult brain and adult liver, respectively. RPE, retinal pigment epithelium; NR, neural retina; NBL, neuroblastic layer; GCL, ganglion cell layer; ONL, outer nuclear layer; INL, inner nuclear layer.

To investigate *mr-s *expression, we first performed whole mount *in situ *hybridization in mouse embryos. No hybridization signal was detected at E9.5, E10.5 and E11.5 with an *mr-s *probe (data not shown). We then investigated the expression of the *mr-s *gene in the developing retina by section *in situ *hybridization (Fig. 1A–G). No significant signal was detected in the developing retina at E13 (Fig. 2A). A weak signal was initially detected in the outer aspect of the neuroblastic layer (NBL), a presumptive photoreceptor layer at E18 (Fig. 2B, arrow). At P3, an *mr-s *transcript was clearly detected in the developing photoreceptor layer (Fig. 2E). At P6, *mr-s *showed peak expression in the photoreceptor layer (Fig. 2F). This pattern correlates with the rapid increase in cells expressing *rhodopsin *and other phototransduction genes between P6-P8 [34-37]. At P9, the intensity of the *mr-s *signal was significantly reduced but was localized to the outer nuclear layer (ONL) (data not shown). Faint expression of *mr-s *mRNA was observed in mature photoreceptors in the adult retina (Fig. 2G).

To determine the tissue specificity of *mr-s *expression, the expression of the *mr-s *gene in various adult tissues was examined by Northern hybridization (Fig. 2H). As a control, P7 retinal RNA was used. Four bands corresponding to 7.2-kb, 4.0-kb, 2.5-kb and 2.2-kb were detected in P7 retina. The 2.2-kb band corresponds to the cDNA characterized in this study. The larger bands, possibly alternative spliced transcripts, have not yet been characterized. The *mr-s *probe did not detect a band in the adult tissues examined, indicating that these tissues do not express *mr-s *at a level comparable to that of the developing retina.

Previous reports revealed that many photoreceptor-specific genes are also expressed in the pineal gland [38]. We examined the expression of *mr-s *transcripts in the whole embryo, whole body, retina, pineal gland, brain, liver and other organs at various stages by RT-PCR (Fig. 2I and data not shown). We amplified PCR fragments of 292 bp and 452 bp with primer pairs for genes encoding mouse *mr-s *and *G3PDH*, respectively. In E13 whole embryo and P0 whole body (except for the eye), no *mr-s *signal was detected. As expected, we observed that *mr-s *is expressed in the P7 and adult pineal gland. In the P7 and adult brain, liver and several other organs, the RT-PCR amplified band of *mr-s *was not detected (Fig. 2I and data not shown). Our data showed that *mr-s *is predominantly expressed in developing photoreceptors and the pineal gland.

### Regulation of *mr-s *transcription by Crx homeodomain protein

**Figure 3:**
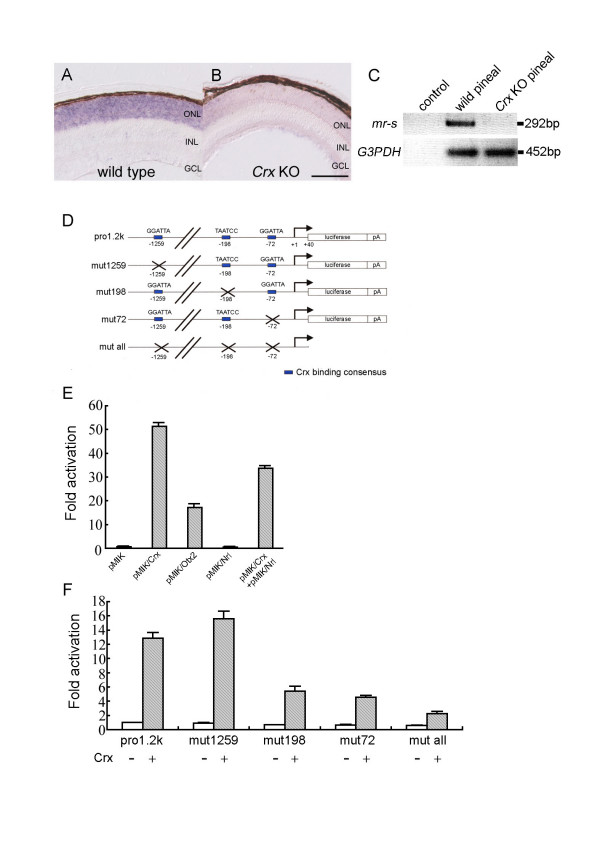
**The transcription of *mr-s *is regulated by Crx. **(A, B) *In situ *hybridization using a probe for mouse *mr-s *was performed on the wild-type (A) and *Crx *KO retinas (B) at P5. *mr-s *expression was drastically reduced in the *Crx *KO retina (B). Scale bar, 100 μm. (C) RT-PCR analysis of total RNAs extracted from the pineal glands of P5 wild-type and *Crx *KO mouse. The upper and lower lanes show PCR products amplified by the primer pairs specific for *mr-s *and *G3PDH *cDNAs, respectively. Water was used for control RT-PCR reaction. (D-F) Crx transactivates *mr-s *transcription. Reporter plasmids for the luciferase assay are shown. Blue boxes represent Crx binding sites (D). Relative activity of the luciferase is indicated when Pro1.2k was co-transfected with *Crx*, *Otx2*, *Nrl*, and *Crx*+*Nrl*, respectively (E). Fold activation is indicated when Pro1.2k, mut1259, mut198, mut72 and mut all were co-transfected with the *Crx *expression vector (Crx+) or the empty vector (Crx-) into HEK293T cells (F). Error bars represent standard error of mean.

Transcription factors Otx2 and Crx are known to be key regulators of retinal photoreceptor development [3,8,9]. Although both *Otx2 *and *Crx *are expressed in developing photoreceptor cells in the embryonic retina, only *Crx *is highly expressed in the postnatal photoreceptors, suggesting that *mr-s *may be regulated by Crx. To test whether Crx regulates *mr-s *transcription, we performed *in situ *hybridization of *mr-s *mRNA on wild-type and *Crx *KO P5 retinas (Fig. 3A, B). In the *Crx *KO retina, the *mr-s *transcript was dramatically reduced (Fig. 3B). This indicates that Crx is essential for transactivation of *mr-s*. Moreover, to test whether the transcription of *mr-s *is also regulated by Crx in the pineal gland, RT-PCR analysis was used to compare transcriptional levels of *mr-s *in P28 wild-type and *Crx *KO pineal gland (Fig. 3C). The results revealed that *mr-s *transcription was significantly downregulated in the *Crx *KO pineal gland. Taken together, these data indicate that *mr-s *transcription is regulated by Crx in the developing photoreceptors and pineal gland.

To further examine whether Crx regulates *mr-s *transcription directly or not, we next performed a luciferase assay using the 1.2-kb proximal promoter region of *mr-s *fused to a luciferase gene as a luciferase reporter (Fig. 3D, Pro1.2k) and the *Crx*, *Otx2*, *Nrl *expression vectors, respectively. This 1.2-kb region of the *mr-s *upstream sequence contains three Crx binding consensus sequences. As shown in Fig. 3E, the luciferase activity was significantly upregulated when the *Crx *or *Otx2 *expression vector was co-introduced with Pro1.2k into HEK293T cells, while the luciferase activity was not altered when the *Nrl *expression vector was co-introduced. A previous report suggested that the transcriptional activity of Crx is augmented with Nrl when the *rhodopsin *promoter was used as a reporter gene [6]. On the other hand, our present data showed that the luciferase gene expression was not upregulated when both *Crx *and *Nrl *expression vectors were co-introduced with Pro1.2k compared to the activity when the *Crx *only expression vector was introduced. This may be due to cell type differences because a cell type of retinal/pineal origin was not used in our luciferase assay. In addition, our present data showed that Otx2, which is reported to have the same binding consensus as Crx, also transactivated *mr-s *expression. As shown in Fig. 2A–F, the expression pattern of *mr-s *correlates with that of *Crx*. In contrast, the transcripts of *Otx2 *are mainly detected in the photoreceptor layer at embryonic stages. Therefore, we concluded that *mr-s *transcription is directly regulated mainly by Crx.

We also constructed reporter vectors in which mutations were introduced at the three Crx binding sites (Fig. 3D, mut1259, mut198, mut72, mut all). Then the *Crx *expression vector was co-introduced with mut1259, mut198, mut72 and mut all, respectively (Fig. 3F). The transactivaton activity by Crx was clearly reduced when mut198 or mut72 was co-introduced. On the other hand, when mut1259 was co-transfected, the transactivation activity by Crx was not altered. These results suggest that the Crx binding sites 72-bp and 198-bp upstream from the transcription initiation site are crucial for the direct regulation of *mr-s *transcription by Crx.

### Self-association of mr-s protein

**Figure 4:**
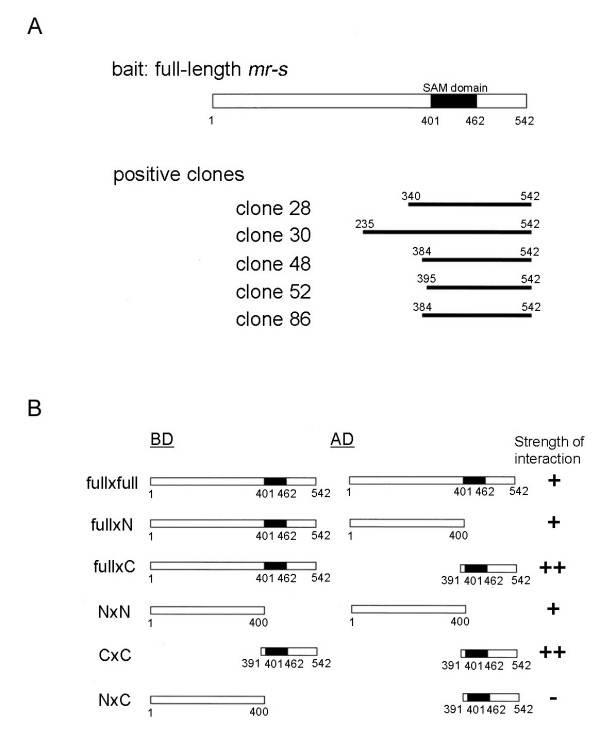
**Summary of yeast two-hybrid screening and GAL4 assay. **(A) Full-length *mr-s *as a bait used in the yeast two-hybrid screening and positive clones are indicated. Note that all of five *mr-s *clones identified in the screening contain SAM domains. (B) Schematic diagram of the mr-s fusion proteins used in the yeast GAL4 assay. Black boxes represent the position of SAM domains. Relative levels of *LacZ *expression are shown on the right, respectively. Note; ++ indicates an intense blue signal visible after 12hr of incubation at 37°C, + indicates a blue signal visible after 24hr of incubation. BD, binding domain; AD, activation domain; full, full-length mr-s; N, N-terminal portion of mr-s (amino acids 1 to 400); C, C-terminal portion of mr-s (amino acids 391 to 542).

SAM domains are known to function as protein-protein interaction modules [15-17]. Although SAM domains can bind to various non-SAM domain-containing proteins, many homo-SAM and hetero-SAM domain interactions have been reported. To investigate whether the SAM domain of the mr-s protein can also function as a protein-protein interaction module, we performed yeast two-hybrid screening using full-length mr-s protein as the bait. Using this bait, we screened the transcriptional activator fusion protein library in which mouse P0-P3 retinal cDNAs were fused to the GAL4 activation domain. The most frequent positive clones (5 out of 28) were cDNA fragments containing the SAM domain of mr-s (Fig. 4A). This result strongly suggests that mr-s protein self-associates through SAM domain-containing regions. We then directly tested this self-association of mr-s protein in yeast. We fused full-length or truncated portions of the mr-s protein to the DNA-binding domain of the yeast transcription factor GAL4 to make bait constructs. We fused full-length or truncated portions of the mr-s protein to the GAL4 transcriptional activation domain to make prey constructs (Fig. 4B). These constructs were transformed into yeast that contain a transgene with GAL4 binding sites upstream of the *lacZ *gene. We found that the full-length mr-s bait construct induced *lacZ *expression with the full-length mr-s prey construct (Fig. 4B, full × full). The N-terminus 400 amino acid (aa) stretch of mr-s, which does not contain a SAM domain, does weakly activate transcription of *lacZ *(Fig. 4B, full × N). The N-terminus 400 aa stretch of mr-s was able to induce transcription of *lacZ *weakly with the same N-terminus 400 aa stretch of mr-s (Fig. 4B, N × N). Although the N-terminus 400 aa mr-s protein weakly activates *lacZ *transcription with the same N-terminus portion, a much stronger activation of *lacZ *expression was observed with a C-terminus portion encoding the 391–542 aa stretch of mr-s (Fig. 4B, full × C, C × C). Our GAL4 assay indicated that the signal when the full-length mr-s was present in both the bait and prey contexts was weaker than when isolated SAM domains were used. This may simply reflect the tendency for the small fusion proteins to enter the yeast nucleus and occupy GAL4 binding sites. Alternatively, the SAM domain may be less accessible for interaction in the full-length protein context as previously reported [39].

**Figure 5:**
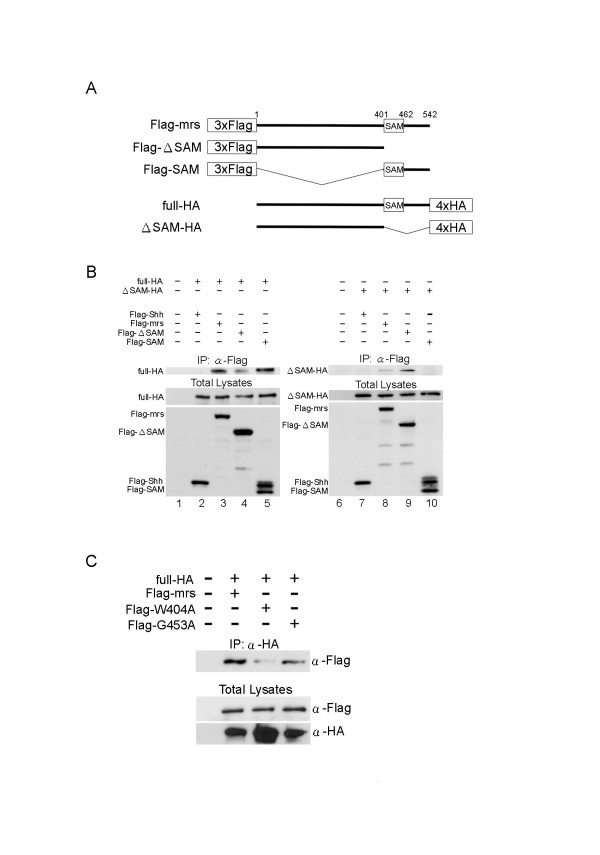
**The mr-s protein can self-associate in mammalian cells. **(A) Schematic drawing of the constructs used for immunoprecipitation assay. HA-tagged or Flag-tagged full-length (amino acids 1 to 542), ΔSAM (amino acids 1 to 400) and SAM (amino acids 401 to 542) regions were inserted into pcDNA3 vector, respectively. (B) The constructs indicated above were transfected into HEK293T cells. Each lane was co-immunoprecipitated by anti-Flag antibody and detected by anti-HA antibody. Input protein lysates are shown in the lower panels. (C) Flag-tagged two site-directed mr-s mutants, Flag-W404A and Flag-G453A were generated and co-transfected with full-HA. Each lane was co-immunoprecipitated by anti-HA antibody and detected by anti-Flag antibody.

To confirm self-association of the mr-s protein in mammalian cells, we next performed co-immunoprecipitation studies in HEK293T cells by co-transfection of HA-tagged full-length/truncated mr-s and Flag-tagged full-length/truncated mr-s (Fig. 5A). As a negative control, we constructed Flag-tagged Sonic hedgehog (Shh) (lane 2 and 7). In accordance with the result of the yeast two-hybrid GAL4 assay, HA-tagged full-length mr-s (full-HA) was co-immunoprecipitated with Flag-tagged full-length mr-s (Flag-mrs) and the Flag-tagged C-terminus portion containing the SAM domain (Flag-SAM), respectively (Fig 5B, lane3 and lane5). We also found a weak co-immunoprecipitation band in co-transfection of full-HA and Flag-tagged N-terminus portion of mr-s (Flag-ΔSAM, Fig. 5B, lane 4). When ΔSAM-HA and Flag-tagged deletion mutants were co-transfected, ΔSAM-HA was co-immunoprecipitated with Flag-mrs and Flag-ΔSAM (Fig. 5B, lane 8 and lane 9), while ΔSAM-HA was not co-immunoprecipitated with Flag-SAM (Fig. 5B, lane 10).

To investigate whether the mr-s protein self-associates mainly through the SAM domain, two site-directed mutations were generated in the SAM domain of mr-s (Fig. 1B, arrows). These mutations alter residues that are conserved in the SAM domain of ph and previous report indicates that these mutations of ph-SAM cause significant reduction in binding activity to the other SAM domain-containing protein, Sex comb on midleg (Scm) (41). Based on this result, we introduced two types of site-directed mutations, which correspond to the mutations introduced in ph protein, into Flag-tagged full-length mr-s (Flag-W404A and Flag-G453A). We found that Flag-W404A binding activity was significantly reduced and Flag-G453A binding activity was also slightly reduced compared to Flag-mrs (Fig. 5C). These results, together with yeast two-hybrid GAL4 assay, indicate that the mr-s protein self-associates strongly through its SAM domain and weakly through the N-terminus portion lacking SAM domain.

### The subcellular localization of mr-s protein in mammalian cells

**Figure 6:**
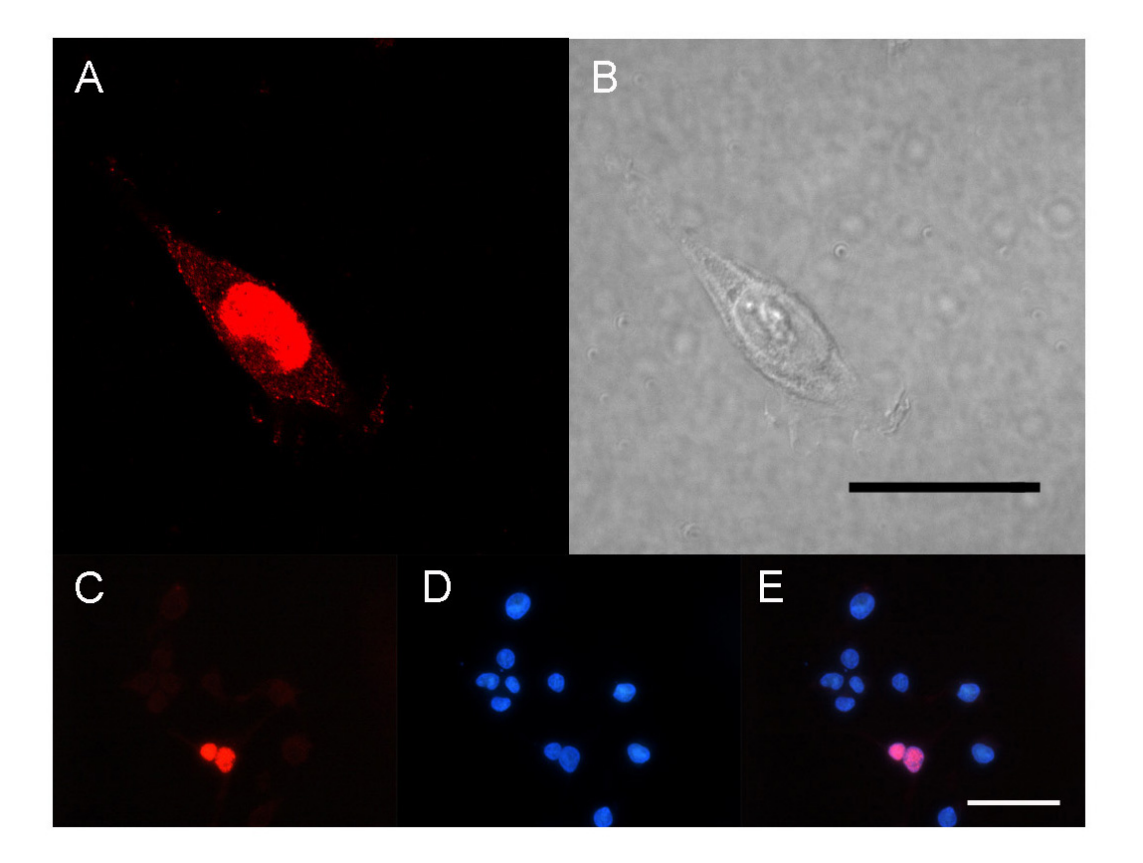
**Subcellular localization of mr-s in mammalian cells. **(A, B) HA-tagged full-length *mr-s *was transfected into HEK293T (B) and detected by anti-HA antibody (A). Scale bar, 20 μm. (C-E) HEK293T cells immunostained with anti-HA antibody (C), DAPI (D), and merged image (E). Scale bar, 50 μm.

The putative nuclear localization signal at the N-terminus of the mr-s protein (Fig. 1A, dashed box) suggests that the mr-s protein localizes in the nucleus. To determine the subcellular localization of mr-s in mammalian cells, we introduced an HA-tagged full-length *mr-s *plasmid into HEK293T cells. Confocal microscopy showed that the mr-s protein localized mainly in the nucleus of HEK293T cells (Fig. 6A, B). To confirm the precise localization of the mr-s protein, full-length mr-s was co-immunostained with DAPI (Fig. 6C–E). These data showed that the mr-s protein localizes preferentially to the nucleus in mammalian cells, supporting the idea that the mr-s protein is involved in transcriptional regulation as are ph and/or TEL.

### The GAL4-mr-s fusion protein functions as transcriptional repressor

**Figure 7:**
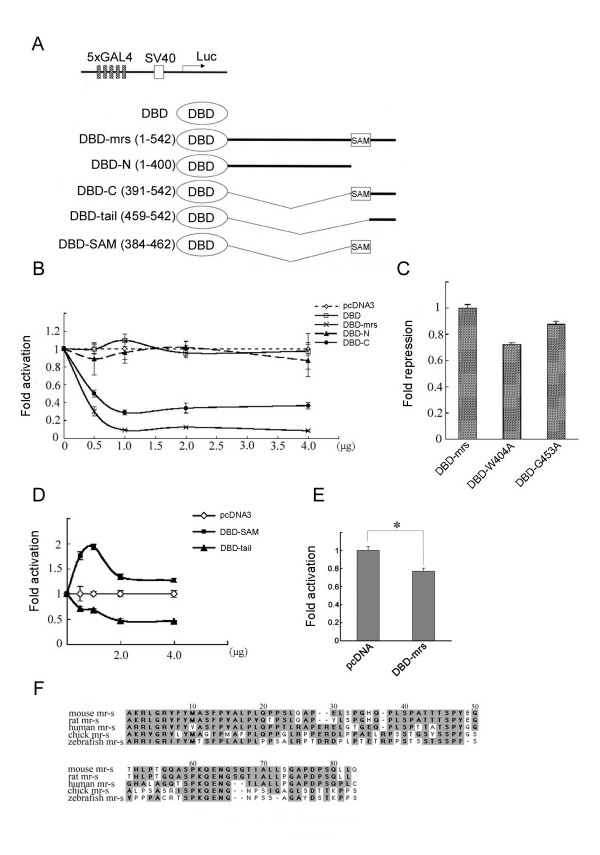
**mr-s fused to GAL4 DNA binding domain functions as a transcriptional repressor in HEK293T cells. **(A) Schematic drawing of the constructs used for the luciferase assay. 5xGAL4-pGL3 reporter plasmid was co-transfected into HEK293T cells with effector plasmids expressing various deletion mutants fused to GAL4-DBD. (B) Various amounts of DBD, DBD-mrs, DBD-N or DBD-C plasmids were transfected with 0.1 μg of 5xGAL4-pGL3 reporter plasmid. The reporter activity in the presence of the pcDNA3 vector (pcDNA3) was designated as 1. Error bars represent standard error of mean. (C) DBD-W404A and DBD-G453A were co-transfected into HEK293T cells with 5xGAL4-pGL3 reporter plasmid. Fold repression was calculated as the fold decrease in luciferase activity compared with DBD-mrs. Error bars represent standard deviation. (D) Various amounts of DBD-tail or DBD-SAM were transfected with 5xGAL4-pGL3 reporter plasmid. Error bars represent standard error of mean. (E) pcDNA3 or DBD-mrs (5 μg) was co-transfected into Y79 retinoblastoma cells with 0.5 μg of 5xGAL4-pGL3 reporter plasmid. The reporter activity in the presence of pcDNA3 was designated as 1. Error bars represent standard deviation. Asterisk marks statistically significant difference (Student's t test: p < 0.03). (F) Alignment of the C-terminal regions for mouse, rat, human, chick and zebrafish mr-s proteins. Conserved amino acid residues are shown with a dark shadow.

A member of PcG proteins, *ph*, does not contain an obvious sequence-specific DNA binding motif (16). Ph functions as a transcriptional repressor through its polymerization and protein-protein interaction with other sequence-specific transcriptional repressors, which can form a higher order chromatin structure. The mr-s protein also does not have an obvious DNA-binding domain. To characterize the biochemical activity of mr-s, we next performed a luciferase assay. We generated effector plasmids, which express various deletion constructs of mr-s fused to the GAL4 DNA-binding domain (Fig. 7A). We first confirmed that full-length mr-s fused to GAL4 DNA binding domain (DBD-mrs) had no effect on the pGL3 promoter plasmid lacking GAL4 binding sites (data not shown). When the 5xGAL4-pGL3 reporter plasmid was co-transfected, DBD-mrs repressed luciferase activity by about 90% in a dose-dependent manner (Fig. 7B). As a control, GAL4 DNA-binding domain (DBD) had no significant effect on the 5xGAL4-pGL3 reporter plasmid. In addition, we confirmed that full-length mr-s without GAL4 DBD had no effect on the same reporter plasmid (data not shown). We next analyzed deletion constructs in which the N-terminus 400 aa stretch of mr-s (amino acids 1 to 400) or the C-terminus portion (amino acids 391 to 542) were fused to the GAL4 DBD (Fig. 7A, DBD-N, DBD-C). While DBD-N had no repressive effect on this reporter activity, DBD-C repressed luciferase gene expression by about 65%. This result suggested to us the possibility that DBD-mrs exerts transcriptional repressive activity via self-association through its SAM domain. To investigate whether the homophilic association of mr-s is required for transcriptional repression, two site-directed mutants, DBD-W404A and DBD-G453A, either of which may reduce self-binding ability, were analyzed (Fig. 7C). Compared to DBD-mrs, DBD-W404A and DBD-G453A had repression activity of 72% and 87%, respectively. While the ability of mr-s self-association partially correlates with transcriptional repressive activity, these mutants do not compromise the ability to repress transcription critically. To determine the regions of mr-s involved in transcriptional repression more precisely, the C-terminus portion was divided into two regions and each was fused to the GAL4 DBD (Fig. 7A, DBD-tail, DBD-SAM). As a consequence, DBD-SAM (amino acids 384 to 462) did not have a repressive effect on the 5xGAL4-pGL3 reporter plasmid. On the other hand, luciferase activity was repressed by 55% when DBD-tail (amino acids 459 to 542) was co-transfected with this reporter plasmid (Fig. 7D). To assess the transcriptional repressive activity of mr-s in cells of retinal origin, we performed similar experiments using human Y79 retinoblastoma cells. The results indicated that DBD-mrs also reduced luciferase activity significantly in Y79 retinoblastoma cells (Fig. 7E). However, luciferase activity was repressed by about 30% in Y79 cells, while it was repressed by 90% in HEK293T cells. This might be due to the difference in transfection efficiency between these cell lines. Another possibility is that intracellular environment in Y79 cells, a retinoblastoma cell line, is insufficient for recapitulating developing photoreceptors. In this study, we did not address the question whether or not our *in vitro *data reflect native mr-s transcriptional repression *in vivo*. However, these *in vitro *experiments using HEK293T and Y79 cells strongly support our hypothesis that mr-s functions as a transcriptional repressor in developing photoreceptors. Our results suggested that the C-terminal region of mr-s (amino acids 463 to 542) is required for transcriptional repression of mr-s and the SAM domain appears to be dispensable for this repressive activity. This C-terminal region of mouse mr-s is highly conserved among species (Fig. 7F). The sequence identity of the region was 93%, 73%, 41% and 40% for rat, human, chick and zebrafish, respectively (Fig. 1D). This strongly suggests that the C-terminal region of mouse mr-s functions as a transcriptional repressive domain in photoreceptor development. However, this region does not contain characteristic amino acid motifs and the mechanism through which the region achieves and/or maintains gene repression remains to be clarified in the future.

Taken together, our findings suggest that DBD-mrs functions as a transcriptional repressor and that the repression activity of mr-s is not due to a homophilic interaction through its SAM domain but to the C-terminal region (amino acids 463 to 542).

## Discussion

In the present study, we identified a novel gene, *mr-s*, which is predominantly expressed in retinal photoreceptors and the pineal gland. The peak of *mr-s *expression in the developing retina is around P6. This expression pattern correlates with the rapid increase of *Crx*, *rhodopsin *and other photoreceptor genes around P6-P8. Around P6, the outer plexiform layer becomes visible and the outer layer of retina separates into two layers, ONL and INL. At the same time, photoreceptors begin to undergo terminal differentiation, forming the outer segment. We therefore hypothesized that *mr-s *is a key molecule in the late development of photoreceptors.

We previously reported that *Otx2 *and *Crx *have a critical role in photoreceptor development and that Otx2 directly regulates *Crx *transcription [3,9]. *In situ *hybridization and RT-PCR showed significant reduction of *mr-s *signal in the *Crx *KO retina and pineal gland. Furthermore, the luciferase assay demonstrated that Otx2 and Crx may directly upregulate the transcription of *mr-s *in mammalian cells. In retinal photoreceptor cells, the *Otx2 *transcripts are not highly expressed at P6-P9, while the *Crx *transcripts are strongly detected around P6. Therefore, our results strongly suggest that *mr-s *transcription is directly regulated by Crx. In the present study, *Nrl*, a photoreceptor-specific transcription factor that is highly expressed in photoreceptors at the postnatal stage, did not affect the transcription of *mr-s*. This finding is actually consistent with the analysis of the *Nrl *KO mouse which was recently reported [40]. The expression profiles of wild-type and *Nrl *KO retinas at P2, P10 and 2 months were analyzed and *mr-s *was not included in 161 differentially expressed genes in the *Nrl *KO retina.

Previous reports suggested that the SAM domain is a protein-protein interaction module. The SAM domain of the mr-s protein is closely related to that of ph and TEL, whose SAM domains can form a helical, head-to-tail polymeric structure and mediate the formation of a higher order chromatin structure. To characterize the biochemical function of mr-s, we performed yeast two-hybrid screening using full-length mr-s as the bait. As a result, the most frequent positive clones (5/28) in the screening were the cDNA fragments containing the SAM domain of mr-s. This strongly suggests that mr-s self-associates through its SAM domain. An immunoprecipitation assay, using two site-directed mutants of the SAM domain of mr-s, demonstrated that the mr-s protein can self-associate through its SAM domain in mammalian cells. While we did not address the question whether the SAM domain of mr-s forms a polymeric structure in the present study, the phylogenetic analysis of SAM domain of mr-s and other SAM domain-containing molecules suggests that mr-s can form head-to-tail polymer and mediate gene silencing by spreading repressive complexes along the chromatin similar to ph and/or TEL. Although our results in the immunoprecipitation assay demonstrated that the N-terminal constructs lacking a SAM domain still interact with each other, these results do not fit into the head-to-tail polymer model. We cannot exclude the possibility that the resulting protein-protein interaction of mr-s is an artifact of the overexpression conditions. The issue of whether or not mr-s forms a polymer awaits future analysis.

A previous report indicated that TEL contains a sequence-specific DNA binding domain, namely the ETS domain, and binds to specific sites via its ETS domain [41]. TEL could serve to nucleate a polymer, which would spread by oligomerization of the SAM domain. In contrast to TEL, ph does not contain an obvious sequence-specific DNA binding motif (16). Therefore, its initial binding to the template may require protein-protein interactions with other sequence-specific transcriptional repressors. The segmentation gene-encoding transcriptional repressors such as Hunchback have a role in recruiting SAM domain-containing PcG proteins, which can spread along the template via polymerization [42]. Since mr-s does not contain obvious DNA binding motifs, we suppose that there is a sequence-specific transcription factor(s) which interacts with mr-s. However, we did not find any transcription factors in the present yeast two-hybrid screening.

We also found that full-length mr-s fused to the GAL4 DNA binding domain (DBD-mrs) functions as a transcriptional repressor. This may support the idea that mr-s is involved in repressive complexes similar to other SAM domain-containing proteins. Our results, however, showed that the self-association of mr-s through its SAM domain is not essential for the transcriptional repressive activity. Polymerization of the SAM domain has been previously reported to be essential for the repressive functions of ph and TEL [43,44]. On the other hand, human lethal(3) malignant brain tumor (H-L(3)MBT) protein, which also contains a SAM domain at the C-terminus, was reported as a transcriptional repressor and the repressor activity of H-L(3)MBT required mainly the presence of the MBT repeats but not the SAM domain [45]. To determine the transcriptional repressor region of the mr-s protein, we performed a luciferase assay using site-directed mutants (DBD-W404A and DBD-G453A). The result showed that the reduced binding ability of self-association partially compromises the transcriptional repressive activity of mr-s (Fig. 7D). However, the repressive effect was more significant when DBD-tail, which does not contain SAM domain, was co-introduced with the 5xGAL4-pGL3 reporter plasmid. Therefore, we conclude that a region from amino acids 463 to 542 is mainly responsible for the repressor activity in the case of mr-s. Evolutionary conservation of the C-terminal 80 aa region of mr-s from zebrafish through human may underlie the functional importance of this region.

Two distinct multiprotein PcG complexes, PRC1 and PRC2, have been identified. PRC2 is involved in the initiation of silencing and contains histone deacetylases (HDACs) and histone methyltransferases, which can methylate histone H3 lysine 9 and 27, marks of silenced chromatin. PRC1, including ph, recognizes the histone H3 lysine 27 mark set by PRC2 and maintains a stable state of gene repression in which PRC1 blocks chromatin remodeling by the trithorax group-related SWI-SNF complex [46,47]. Therefore, the mechanism of repression by PRC2 is thought to be HDAC-dependent while the mechanism of repression by PRC1 appears to be HDAC-independent. Our luciferase assay also showed that transcriptional repression of DBD-mrs was not affected by the addition of various concentrations of trichostatin A, a potent HDAC inhibitor (data not shown). Therefore, we speculate that the mechanism of repression of mr-s may be HDAC-independent and more similar to that of PRC1 complex.

In the present study, the biochemical experiments demonstrate that the mr-s protein functions as a transcriptional repressor and possibly down-regulates the spatial and temporal expression of the target genes during retinal photoreceptor development. Our data also revealed that the repressive activity of mr-s is mainly due to the C-terminal region (amino acids 463 to 542). However, downstream targets of *mr-s *still remain unclear. *In situ *hybridization showed that the peak of *mr-s *expression is around P6, when retinal photoreceptors undergo terminal differentiation. We hypothesize that the target genes of mr-s might be non-photoreceptor genes. In this case, mr-s may suppress the expression of non-photoreceptor genes in rod and cone photoreceptors. There may be another possibility that mr-s is involved in cell fate determination of rod photoreceptors versus cone photoreceptors. While cone photoreceptors are born during the early embryonic stages of mouse retinogenesis, rod photoreceptors are born primarily in the late embryonic and early postnatal period [48]. The expression pattern of *mr-s *may suggest that *mr-s *is expressed in rod photoreceptors but not in cone photoreceptors as well as Nr2e3, which is known as a transcriptional repressor and is thought to down-regulate cone photoreceptor-specific genes. To clarify the biological function of *mr-s*, analysis of mice with targeted disruptions of *mr-s *will be very important.

## Conclusion

Here we identified mouse *mr-s*, which is predominantly expressed in retinal photoreceptors and the pineal gland. *mr-s *is evolutionarily conserved from zebrafish through to human, suggesting a significant role of *mr-s *in photoreceptor development. Our present data suggest that mr-s protein localizes in the nucleus and can self-associated mainly through the SAM domain. Moreover, mr-s protein fused to GAL4 DBD functions as a transcriptional repressor. The repressive activity of mr-s is due to its C-terminal region (amino acid 463 to 542). Taken together, mr-s is a novel repressor molecule possibly involved in the development of retinal photoreceptors and pineal gland.

## Methods

### Isolation of mouse *mr-s *cDNA

We used the bioinformatics method Digital Differential Display (NCBI, UniGene) to screen novel mouse genes expressed preferentially in the retina. Some of the clusters in the UniGene database were mainly from mouse retinal cDNAs. One clone in these clusters (#Mm. 246385) has a weak homology with the *polyhomeotic *family genes. A 735-bp cDNA fragment of this clone, encoding amino acids 140–384 was amplified by RT-PCR from mouse P0 retinal cDNA. This fragment was used as the probe for library screening, *in situ *hybridization and Northern hybridization. A mouse P0-P3 retinal cDNA library was screened using this mouse cDNA fragment. Positive bacteriophage clones were isolated and the full-length *mr-s *fragment was inserted into pBluescriptII (Stratagene). DNA sequencing was performed on both strands by the cycle sequencing method. The nucleotide sequence for mr-s gene has been deposited in the GenBank database under GenBank Accession Number #AY458844.

### In situ hybridization

The 735-bp cDNA fragment of *mr-s *amplified by RT-PCR was used as a probe for *in situ *hybridization. *In situ *hybridization was performed as described previously [49].

### Cell culture and transfection

HEK293T cells were maintained at 37°C in Dulbecco's modified Eagle's medium (DMEM) supplemented with 10% fetal bovine serum (Sigma), 100 IU/ml penicillin and 100 μg/ml streptomycin. Transient transfection of HEK293T cells was carried out using calcium phosphate method or Fugene6 transfection reagent (Roche). Y79 retinoblastoma cells were maintained in Iscove's modified Dulbecco's medium with 4 mM L-glutamine adjusted to contain 1.5 g/L sodium bicarbonate, 20% FBS. Transient transfection was carried out using TransIt LT1 (Mirus).

### Northern-blot analysis

RNA was extracted from the tissues of adult mice using Trizol (Invitrogen). A 5 μg of total RNA was electrophoresed in a 1.0% agarose-formaldehyde gel and transferred to a nylon membrane (Zeta-Probe GT, Bio-Rad). The 735-bp cDNA fragment encoding the SAM domain of *mr-s *was used as a probe for hybridization. Hybridization was performed according to the manufacturer's protocol. Washes with increasing stringency were performed, the last being at 50°C in 0.1× standard saline citrate/0.1% sodium dodecyl sulfate (SDS).

### RT-PCR analysis

Total RNA was isolated from each tissue using Trizol. A 1 μg of total RNA was reverse transcribed using SuperscriptII (Invitrogen). RT-PCR primers, which span introns, for detection of *mr-s *cDNA were 5'-TGTCCAGCCCAGCCAACCCAAGGAGACGACA-3' and 5'-TGTGGTCTCCTCATCAGTGAAGA-3'. Product size was 292 bp (positions 965–1256 of Genbank Accession Number #AY458844). Primer pairs for mouse *G3PDH *were 5'-ACCACAGTCCATGCCATCAC-3' and 5'-TCCACCACCCTGTTGCTGTA- 3' which amplified a 452-bp product (positions 587–1038 of Genbank Accession Number #BC85275).

### Yeast two-hybrid screening and GAL4 assay

We carried out yeast two-hybrid experiments using the MATCHMAKER GAL4 two-hybrid system 3 (BD Bioscience) as recommended by the manufacturer. We cloned the full-length mr-s into the pGBKT7 vector and used it to screen a library of mouse P0-P3 retinal cDNAs in the pGADT7 vector. Transformants that conferred growth were picked, isolated and re-introduced with the bait into AH109 to confirm interaction. Plasmid DNA was isolated from yeast using RPM Yeast Plasmid Isolation Kit (Qbiogene). In order to confirm the protein interactions, single colonies were picked and grown individually in synthetic complete media lacking leucine, tryptophan and containing X-gal. After 4–5 days, X-gal positive clones were selected and analyzed. To test self-interaction of mr-s, we subcloned the full-length, N-terminus (amino acids 1–400) and C-terminus (amino acids 391–542) of mr-s into pGBKT7 and pGADT7 vectors. We assessed interactions by scoring blue color on plates of medium containing X-gal.

### Immunoprecipitation assay

For immunoprecipitation assay, 4× hemagglutinin (HA) or 3× Flag tagged cDNA fragment encoding full-length *mr-s *(full-HA and Flag-mrs), a cDNA fragment encoding amino acids 1–400 (ΔSAM-HA and Flag-ΔSAM), and a cDNA fragment encoding amino acids 400–542 (Flag-SAM) were subcloned into the pcDNA3 (Invitrogen) expression vector. We also constructed two site-directed mutants, Flag-W404A and Flag-G453A, using PCR. Each of these mutations was also introduced into Flag-mr-s. We transfected HEK293T cells with 5 μg of plasmid DNA per 6 cm dish by calcium phosphate method. Approximately 48 hr after transfection, cells were harvested in immunoprecipitation buffer (50 mM Tris-HCl [pH 7.5], 1 mM EDTA, 2 mM MgCl_2_, 150 mM NaCl, 1 mM phenylmethylsulfonyl fluoride [Wako], 1% Nonidet P-40, 10% glycerol) in the presence of protease inhibitor cocktail tablets (Roche). For each reaction, 1 mg of cell lysate was mixed with 0.5 μg of anti-Flag antibody (SIGMA, F3165) and 15 μl of protein G-Sepharose (Amersham) on a rotating wheel at 4°C for 2 hrs. Protein concentration was determined by the BCA protein assay system (Pierce). The beads were then washed three times with immunoprecipitation buffer and followed by three times with wash buffer (50 mM Tris-HCl [pH 7.5], 150 mM NaCl, 20 mM MgCl_2_). Proteins were then boiled for 5 min in SDS sample buffer (1% SDS, 1 mM Tris [pH 6.8], 40 mM DTT, 4% glycerol, 0.01% pyronine Y). The supernatants were fractionated by sodium dodecyl sulfate-polyacrylamide gel electrophoresis (SDS-PAGE) and transferred to the nitrocellulose membrane (Trans-Blot Transfer Medium, Bio-Rad). Western blotting was performed with rabbit polyclonal anti-HA antibody (Santa Cruz, #sc-805). Signals were detected with horseradish peroxidase- conjugated goat anti-rabbit IgG and ECL plus Western Blotting Detection System (Amersham).

### Subcellular localization analysis

We transfected the plasmid encoding HA-tagged full-length mr-s into HEK293T cells, seeded cells on coverslips coated with collagen 48 hr after transfection, fixed with 4% paraformaldehyde in phosphate-buffered saline (PBS) at room temperature for 20 min, and washed with PBS. Then we permeabilized cells in PBS containing 0.1% Triton X-100 for 15 min, washed again with PBS, and incubated in PBS containing 0.1% bovine serum albumin (BSA) for 30 min at room temperature. We incubated cells with anti-HA antibody overnight at 4°C (1:200 in PBS containing 0.2% BSA). The following day, we washed cells three times with PBS and incubated with a Cy3-conjugated goat IgG against rabbit IgG (1:400 in PBS, Jackson ImmunoReseach Laboratories) for 30 min. We rinsed cells three times with PBS, followed by observation using a confocal microscope FV300 (Olympus) equipped with a 60× objective lens.

### Luciferase assay

For transcriptional analysis of the 1.2-kb promoter region of mr-s, we constructed a reporter plasmid (pro1.2k) by subcloning a 1.2-kb upstream genomic fragment of *mr-s *gene into a pGL3 luciferase reporter plasmid (Promega). The expression vectors were constructed by subcloning full-length *Crx*, *Otx2, Nrl *genes into a pMIK expression vector (a gift from Dr. K. Maruyama), respectively. For the "mut1259" vector, the Crx binding site at the -1259 bp position was mutated by replacing GGATTA with AGATCT. For the "mut198" vector, the Crx binding site at the -198 bp position was mutated by replacing TAATCC with GAATTC. For the "mut72" vector, the Crx binding site at the -72 bp position was mutated by replacing GGATTA with GAATTC. For the "mut all" vector, all of three Crx binding sites were mutated. 5xGAL4-pGL3 Control reporter plasmid, which contains five copies of the GAL4 DNA recognition sequence positioned immediately upstream of SV40 minimal promoter, was kindly gifted from Dr. T. Noguchi and used for analysis of the transcriptional activity of mr-s [50]. To generate effector plasmids, various deletion fragments were produced by PCR reaction and subcloned into pGBKT7 vector, respectively. These fragments were digested with the sequence, which encodes GAL4 DNA binding domain, and inserted into pcDNA3. We transfected 0.1 μg of reporter plasmid DNA and 2 μg of the expression vector DNA per 6 cm dish into HEK293T cells using Fugene6 transfection reagent. We analyzed luciferase activity 48 hr after transfection.

## List of abbreviations

Crx, cone-rod homeobox; Otx2, orthodenticle-related homeobox 2; TRβ2, thyroid hormone receptor beta 2; Nrl, neural retina leucine zipper; Nr2e3, nuclear receptor subfamily 2 group E member 3; EST, expressed sequence tag; EphB2, ephrin receptor B2; EphA4, ephrin receptor A4; TEL, translocation ETS leukemia.

## Authors' contributions

TI and KT performed most of the experiments, interpreted the data and wrote the first draft of the manuscript. AF prepared retinal tissues and their sections of wild-type and Crx mutant mice. CK performed luciferase assay using Y79 retinoblastoma cells. YT, MA and TF have made substantial contributions to conception and design of the experiments and drafting of the manuscript. All authors read and approved the final manuscript.
